# Improvement of Tardive Dyskinesia during Mindfulness Meditation

**DOI:** 10.3390/neurolint13030043

**Published:** 2021-08-30

**Authors:** Maria Angela Santoro, Isolde English, Idil Sezer, Mickael Amagat, Frank Ly, Edouard Chaneac, Patricia Cailliez, Hugo Bottemanne

**Affiliations:** 1 Department of Psychiatry, Pitié-Salpêtrière Hospital, DMU Neuroscience, Sorbonne University, Assistance Publique-Hôpitaux de Paris (AP-HP), 75651 Paris, France; Maria.angela.santoro@aphp.fr (M.A.S.); Isolde.english@gmail.com (I.E.); Mickael.amagat@aphp.fr (M.A.); Frank.ly@aphp.fr (F.L.); 2 Paris Brain Institute-Institut du Cerveau (ICM), Sorbonne University/CNRS/INSERM, UMR 7225/UMRS 1127, 75651 Paris, France; Idil.sezer@aphp.fr; 3 Department of Psychiatry, Bicêtre Hospital, Assistance Publique-Hôpitaux de Paris (AP-HP), 94270 Paris, France; Edouard.chaneac@aphp.fr (E.C.); Patricia.cailliez@aphp.fr (P.C.); 4 Department of Philosophy, SND Research Unit, Sorbonne University, UMR 8011, 75005 Paris, France

**Keywords:** dystonia, dyskinesia, mindfulness-based cognitive therapy, mindfulness meditation, movement disorder, psychiatry

## Abstract

Background: We report the case of a patient presenting with orofacial tardive dyskinesia (TD), following administration of a first-generation antipsychotic (Loxapine). Intervention: Four weeks of repeated sessions of mindfulness-based cognitive therapy (MBCT) and mindfulness-based stress reduction (MBSR) protocols were administered, with TD hetero-quantified before and during each session via the Abnormal Involuntary Movement Scale (AIMS). Results: The dyskinesia ameliorated quantitatively and qualitatively (1) during each session, and (2) at resting conditions in the long term. During some sessions, after which patients’ compliance was auto-evaluated as maximal, complete arrest of the TD was observed. Hypothesis and Conclusion: We suggest mindfulness meditation as a novel adjunctive therapeutic approach for tardive dyskinesia, and invite for further clinical and neurological investigations.

## 1. Introduction

Millions of people in the world are, nowadays, treated at an increasing rate with antipsychotics. This is known to be among the causative factors eliciting tardive dyskinesia in a subset of patients ranging from 20% to 50% [1,2]. Tardive dyskinesia is defined by the DSM5 as a disorder of movement emerging after short- or long-term usage of medications, persisting at least 1 month after discontinuation of drug usage. It is a syndrome characterized by a series of unstoppable, stereotyped movements, more frequently of the orofacial muscles, which dramatically impairs patients’ quality of life [3]. Although we don’t exactly know yet the neurological mechanisms implied, some evidence suggests that super sensitivity of D2 receptor, caused by chronic D2 receptor blockade [4] in the motor striatum (particularly by first-generation antipsychotics), as well as other genetic and environmental factors (age, polypharmacotherapy, etc.), might be involved [5].

Treatment of this syndrome remains challenging without strong evidence of efficacy nor consensus among current approaches. Conventional drug strategies such as cholinergic drugs, gamma-aminobutyric acid agonists (baclofen, progabide) or calcium channel blockers are generally ineffective against dyskinesia [6]. Cholinergic drugs such as deanol, lecithin and meclofenoxate can even cause side effects. In addition, the prescription of anticholinergic drugs (such as procyclidine or isocarboxacid) to reduce the side effects of motor symptoms is probably a factor in the aggravation of dyskinesia. There is evidence from animal experiments that anticholinergic drugs could cause tardive dyskinesia [7]. Recently, some interest has arisen around neuropsychological interventions such as mindfulness meditation because of its inexpensiveness and the general benefits elicited in the psychiatric patients’ population; these practices have already been shown to improve symptomatology in dystonia [8] and may also have a role in iatrogenic dyskinesia, particularly that which is L-dopa induced [9]. These new non-drug approaches are well tolerated by patients, do not cause serious adverse effects and could have a place in therapeutic strategies against dyskinesia induced by neuroleptics [9].

We report here the case of a radical improvement obtained via mindfulness meditation, in a patient suffering from tardive dyskinesia after first-generation antipsychotic treatment, and we discuss possible mechanisms associated with this enhancement.

## 2. Case Report

We report the case of a sixty-year-old male patient suffering from severe recurrent anxio-depressive disorder with hypochondriac dominant features, who, after the introduction of Loxapine, developed some major tardive dyskinesia (TD) involving the lips, jaw and perioral muscles.

The patient’s first major depressive episode was diagnosed in his thirties: an outpatient treatment starting with Fluoxetine 20 mg/per day was proposed but the patient was lost at follow-up. He was hospitalized in his mid-forties for scenarized suicidal thoughts in the context of a major depressive episode with major hypochondriac features; diagnosis of a recurrent major depressive disorder was established. Fluoxetine, which he was still taking, was substituted with Paroxetine 40 mg/per day and then with Sertraline at 200 mg/per day, which, together with the beginning of cognitive behavioural therapy, proved its efficacy and allowed for patient dismission and outpatient follow-up.

Two further hospitalizations were required in the last two years for pharmacological therapy adjustment because of an upsurge in hypochondriac anxio-depressive symptomatology. During the second-to-last hospitalization, the patient’s symptomatology required the beginning of an antipsychotic therapy with an anxiolytic and ideolytic aim, started with Loxapine 150 to 300 mg/per day. The patient did not show signs of extrapyramidal or poor motor tolerance during the administration of Loxapine. The patient presented hypochondriac delusions, congruent with mood, motivating the prescription of Risperidone 2 mg/per day. Sertraline was gradually substituted by Venlafaxine up to 300 mg/day and lithium introduced at 800 mg/day, corresponding to a serum lithemia of 0.7 mmol/L. The clinical improvement made it possible to stop Loxapine after two months of treatment. The patient was dismissed for anxio-depressive symptomatology regression, and addressed to our unit in order to build up to a societal reinsertion project.

He began to present facial tardive dyskinetic movements 3 months after stopping treatment, involving the lips, jaw and perioral muscles, only disappearing during patient sleep. A first assessment of patient’s dyskinesia using the Abnormal Involuntary Movement Scale (AIMS) [10] gave us a Total Score (TS) of 12 (sum of AIMS items 1 to 7) and an Overall Severity Index (OSI) of 3 (AIMS item 8), causing a level of awareness and distress (A&D) considered by the patient themself of 4 (AIMS item 10). Following the patient’s daily progressions, it was noticed that his general stress level reflected quantitatively and qualitatively on the dyskinesia. For this reason, mindfulness-based interventions, such as mindfulness-based cognitive therapy (MBCT), and mindfulness-based stress reduction (MBSR) strategies were employed. As a matter of fact, MBCT and MBSR are already known to reduce the levels of stress hormones [11], to also reduce anxiety [12] and to improve mood symptoms [13]. TD was hetero-quantified through the AIMS before and during any meditation session by a trained operator, in an observational manner.

**Figure 1:**
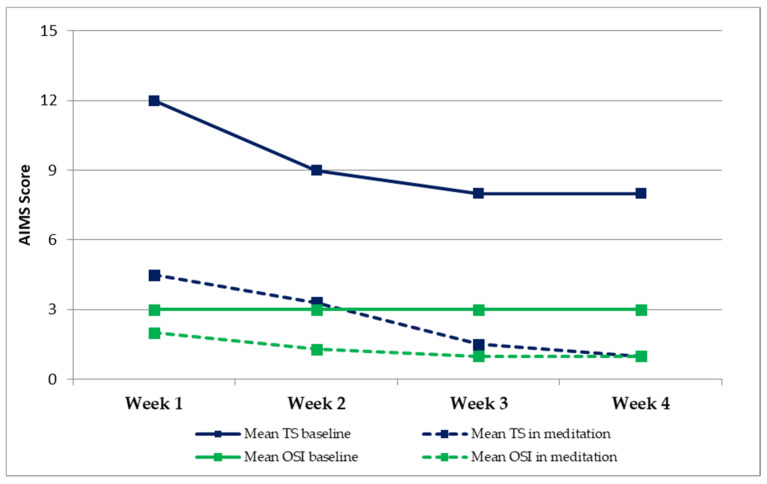
Baseline vs. in-meditation AIMS Total Score (TS) and Overall Severity Index (OSI). AIMS: Abnormal Involuntary Movement Scale. TS: Total score (equal the sum of the AIMS scoring given at the items from 1 to 7). OSI: Overall Severity index (equal the AIMS scoring given at the item 8).

We noticed a generalized reducing trend of the TD over the weeks with a ΔTS between the first and the fourth week of 4 points at baseline condition, as well as a significant intra-intervention decrease in severity and number of TD, with a maximal TS decrease of 7.5 (Figure 1). During the first week, the mean TS before sessions = 12, the mean TS during sessions = 4.5, and Δ = 7.5; the mean OSI before sessions = 3, the mean OSI during sessions = 2, and Δ = 1. During the second week, the mean TS before sessions = 9, the mean TS during sessions = 3.3, and Δ = 5.7; the mean OSI before sessions = 3, the mean OSI during sessions = 1.3, and Δ = 1.6. During the third week, the mean TS before sessions = 8, the mean TS during sessions = 1.5, and Δ = 6.5; the mean OSI before sessions = 3, the mean OSI during sessions = 1, and Δ = 1.5. During the fourth week, the mean TS before sessions = 8, the mean TS during sessions = 1, and Δ = 7; the mean OSI before sessions = 3, the mean OSI during sessions = 1, and Δ = 1.5 (Table 1). AIMS scoring was significantly different between before-meditation and intra-meditation observations (t63 = 7, *p* < 0.01, paired *t* tests).

Dyskinesia decrease was also objectivised via an EEG with perioral muscles electromyography, performed at the end of the four weeks, with an intra-recording meditation session, where the electromyography recording showed concordance with the AIMS scoring: frequency, amplitude and width of the perioral muscles’ contraction waves decreased consistently during the whole closed eyes, relaxed state. Those three parameters (frequency, amplitude and width) all increased each time the patient was asked to re-emerge from the meditative state.

## 3. Discussion

We report here a significant and prolonged clinical improvement of acquired tardive dyskinesia after antipsychotic treatment, following treatment by mindfulness-based cognitive therapy (MBCT) and mindfulness-based stress reduction (MBSR). Research conducted on mindfulness-based interventions suggests that mindfulness meditation produces delta, theta, alpha and beta 1 activity waves, and minimizes incoming signals from immediate surroundings and increases the ability of information processing, particularly related to current states of body [14]. In addition, eye closure and maintenance of a relaxed state are known factors eliciting the insurgence of brain alpha waves, recorded on human brain EEG as a consequence of neuronal discharge synchronization.

The acute clinical improvement during the MBT session can be explained using many hypotheses; in particular, the role of distractibility and attentional focus, which could participate in improving dyskinesia. However, this explanation does not seem sufficient to us, given the prolongation of the effects of MBT on dyskinesias well after the sessions. The improvement in abnormal motor movements during mindfulness-based therapy (MBT) has already been documented [8], but the mechanism associated with prolonged improvement after treatment remains a mystery.

A first hypothesis would be to consider the effect of MBT on the patient’s anxiety level, assuming that the improvement in anxiety may have participated in a decrease in dyskinesias. Studies suggest a link between abnormal motor movements and environmental stress, in particular, a worsening of pre-existing motor abnormalities in stressful situations [15]. A second hypothesis would be to consider the effect of MBT on brain connectivity, a promising field of research. We can, for example, assume that neuronal electrical synchronisation might overwhelm some subcortical non-vital pathways, impeding non-controlled organized discharge, in particular, those leading to stereotyped movements, notably dyskinesia. In the long term, we suggest there is a stabilization of functional neuronal pathways at the expense of others (dyskinetic pathways), as a consequence of the repeated firing of focused attention circuitries, which may induce the creation of reflex negative pathways and may reduce the dyskinetic pathways.

Another possible explanation could be due to large-scale modulation of brain networks mediated by mindfulness meditation. The remodulation of resting-state brain networks, meaning anatomically distinct regions co-activated for a function, could explain the long-lasting therapeutic effects for the patient. The modulation of brain networks, such as the central executive network implicated in attentional processes and the default mode network implicated in internally focused processes [16], could explain the decreased TD through improved attention control and emotion regulation [17]. Indeed, for the latter, the regulation of negative emotions generated by TD symptoms could in part explain the alleviation of symptoms, notably of anxious symptoms. Overall, although its mechanisms remain to be elucidated, mindfulness practice has been extensively linked to therapeutic effects in various clinical populations [18]

Further research using electrophysiology and neuroimaging is needed to understand the effect of MBT on abnormal movements and, in particular, on neuroleptic-induced dyskinesia. Specifically, resting-state functional connectivity (RSFC) methods and structural neuroimaging methods can help us explore how mindfulness meditation can have an impact on neural plasticity [19,20,21]. Parallelly, previous electrophysiological research has suggested long-lasting changes in the resting electroencephalogram patterns during mental practice [22], which is very encouraging for future explorations. This research could be carried out on large samples given the large number of patients with these symptoms in psychiatric hospitals and in outpatient psychiatric care.

## 4. Conclusions

We present a case of tardive dyskinesia improvement during mindfulness meditation. We hypothesized the possibility of long-term instauration of neuronal circuits able to overwhelm those inducing the TD. On a larger scale, we hypothesize the remodulation of brain networks modifying attentional processes and regulating the negative affects concomitant to TD. Little data is available on the neurological mechanisms underlying TD, and the specific neurological mechanisms of mindfulness meditation still remain to be understood. However, there is, interestingly, some proof that it might cause neuroplastic changes [17], and these changes may be involved in the long-term efficacy of MBT against these unwanted motor symptoms.

With regard to this limited knowledge, the above-mentioned proposed mechanisms are only speculative, but we encourage further research for neurobiological explanations, as well as research for greater data strength via protocolized and standardized mindfulness interventions. We present here the results of a case report on a single patient. These results must therefore be considered with caution, and deserve to be replicated on a larger scale. If these findings could be confirmed and validated through a group study bearing statistically significant numerosity, the understanding of the mechanism would be of help in the development of more targeted interventions with probably more stable efficacy, possibly opening further therapeutic horizons.

## Figures and Tables

**Table 1:** AIMS scoring divided per intervention weeks.

	Mean TS Baseline	Mean TS in Meditation	Δ TS	Mean OSI Baseline	Mean OSI in Meditation	Δ OSI
Week 1	12	4.5	7.5	3	2	1
Week 2	9	3.3	5.7	3	1.3	1.6
Week 3	8	1.5	6.5	3	1	1.5
Week 4	8	1	7	3	1	1.5

AIMS: Abnormal Involuntary Movement Scale. TS: Total score (equal the sum of the AIMS scoring given at the items from 1 to 7). OSI: Overall Severity index (equal the AIMS scoring given at the item 8).
